# A systemic approach to identify non-abundant immunogenic proteins in Lyme disease pathogens

**DOI:** 10.1128/msystems.01087-23

**Published:** 2023-12-11

**Authors:** Ozlem Buyuktanir Yaş, Adam S. Coleman, Rachel M. Lipman, Kavita Sharma, Sajith Raghunandanan, Fuad Alanazi, Vipin S. Rana, Chrysoula Kitsou, Xiuli Yang, Utpal Pal

**Affiliations:** 1 Department of Microbiology, Faculty of Veterinary Medicine, Ondokuz Mayis University, Samsun, Turkey; 2 Department of Veterinary Medicine, University of Maryland, College Park, Maryland, USA; 3 Department of Microbiology and Immunology, Indiana University School of Medicine, Indianapolis, Indiana, USA; 4 Virginia-Maryland College of Veterinary Medicine, College Park, Maryland, USA; Drexel University, Berwyn, Pennsylvania, USA

**Keywords:** *Borrelia burgdorferi*, Lyme disease, mutagenesis

## Abstract

*Borrelia burgdorferi*, the pathogen of Lyme disease, differentially produces many outer surface proteins (Osp), some of which represent the most abundant membrane proteins, such as OspA, OspB, and OspC. In cultured bacteria, these proteins can account for a substantial fraction of the total cellular or membrane proteins, posing challenges to the identification and analysis of non-abundant proteins, which could serve as novel pathogen detection markers or as vaccine candidates. Herein, we introduced serial mutations to remove these abundant Osps and generated a *B. burgdorferi* mutant deficient in OspA, OspB, and OspC in an infectious 297-isolate background, designated as *Osp^ABC^*^−^ mutant. Compared to parental isolate, the mutant did not reflect growth defects in the cultured medium but showed differential mRNA expression of representative tested genes, in addition to gross changes in cellular and membrane protein profiles. The analysis of differentially detectable protein contents of the *Osp^ABC^*^−^ mutant, as compared to the wild type, by two-dimensional gel electrophoresis followed by liquid chromatography-mass spectrometry, identified several spirochete proteins that are dominated by proteins of unknown functions, as well as membrane transporters, chaperons, and metabolic enzymes. We produced recombinant forms of two of these represented proteins, BBA34 and BB0238, and showed that these proteins are detectable during spirochete infection in the tick-borne murine model of Lyme borreliosis and thus serve as potential antigenic markers of the infection.

**IMPORTANCE:**

The present manuscript employed a systemic approach to identify non-abundant proteins in cultured *Borrelia burgdorferi* that are otherwise masked or hidden due to the overwhelming presence of abundant Osps like OspA, OspB, and OspC. As these Osps are either absent or transiently expressed in mammals, we performed a proof-of-concept study in which their removal allowed the analysis of otherwise less abundant antigens in OspABC-deficient mutants and identified several immunogenic proteins, including BBA34 and BB0238. These antigens could serve as novel vaccine candidates and/or genetic markers of Lyme borreliosis, promoting new research in the clinical diagnosis and prevention of Lyme disease.

## OBSERVATION

**Fig 1:**
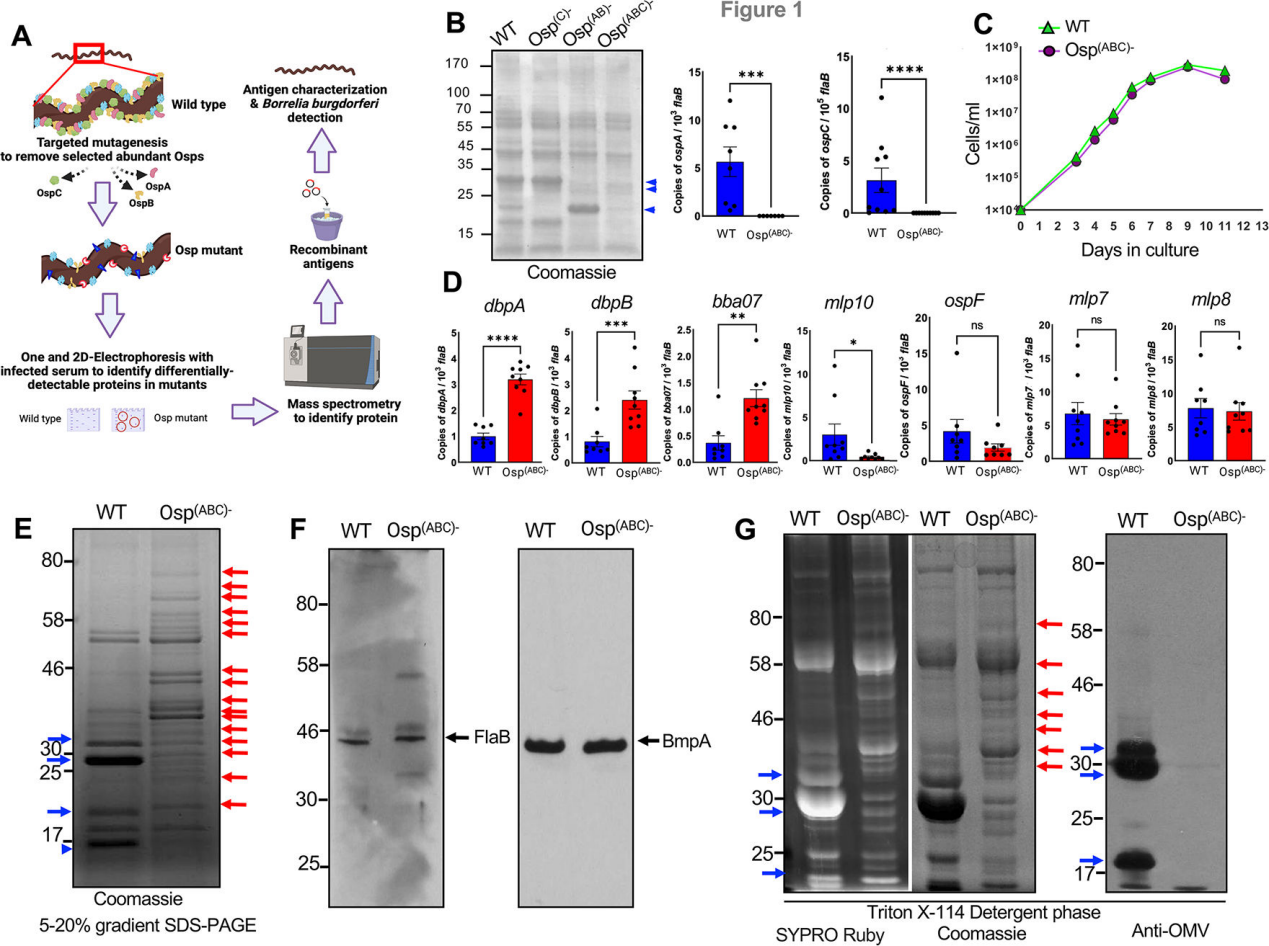
Generation and characterization of the OspABC-deficient mutant. (**A**) Flowchart of the strategy of identifying non-abundant proteins in cultured *B. burgdorferi* cells. (**B**) *Osp^ABC^*^−^ mutant does not produce OspA, OspB, or OspC. *B. burgdorferi* wild-type strain (WT), the *ospC* mutant (12), the *ospAB* mutant (13), and the *Osp^ABC^*^−^ mutant were grown to 5 × 10^7^ spirochetes/mL, and whole cell lysates were subjected to SDS-PAGE and stained with Coomassie blue stain (leftmost panel). The bands corresponding to OspA, OspB, and OspC are indicated by arrows. The two right panels show the real-time quantitative reverse transcription PCR (RT-qPCR) analysis of *B. burgdorferi ospA* and *ospC* levels, as normalized to *flaB* transcripts. Results represent three independent experiments, where quantitative data are shown as individual data points; error bars show the mean ± SD (*n* = 10). ***P < 0.0003; ****P < 0.0001. (**C**) Growth of *Osp^ABC^*^−^ mutant in BSK-II medium was not impaired. The wild type and mutant isolates were grown in the medium at 34°C. Spirochetes were enumerated daily using dark-field microscopy. Data are presented as the mean values ± standard deviation from three biological replicates. (**D**) Expression of target genes in spirochetes. RT-qPCR analysis of *B. burgdorferi* transcript levels, as normalized to *flaB*. Results represent three independent experiments, where quantitative data are shown as individual data points; error bars show the mean ± SD (*n* = 10). Significant differences between samples were determined using the Student’s two-tailed *t*-test using GraphPad prism 9.0. *P < 0.01; **P < 0.002; ***P < 0.001; ****P < 0.0001. (**E**) Whole cell protein profiles in *B. burgdorferi*. Lysates (25 µg/lane) from the wild type and mutant cells were resolved in a large gel (16 cm) using 5–20% gradient SDS-PAGE and stained with Coomassie blue. Proteins that were differentially more detectable in the mutant are indicated by red arrows, while ones in the wild type (including OspA, OspB, and OspC) are shown by blue arrows. (**F**) Production of FlaB and BmpA protein in spirochetes. Western blot analyses of whole cell lysates (5 µg/lane) from wild type and mutant cells using anti-FlaB and anti-BmpA are shown (arrows). (**G**) Analysis of detergent phase proteins in spirochetes. Triton-X-114 detergent extractable proteins from the wild type and mutant cells (30 µg/lane) were resolved using 5–20% gradient SDS-PAGE and stained using either SYPRO Ruby (left panel) or Coomassie blue (center panel). The rightmost panel represents immunoblot analysis of the gel using anti-OMV antibodies. Similar to panel E, more detectable protein bands in the mutant or wild type cells are indicated by red or blue arrows, respectively.

The most common *Ixodes* tick-transmitted infection in many regions of the United States and Europe is Lyme disease or Lyme borreliosis, caused by *Borrelia burgdorferi sensu lato* spirochetes (1). The disease is reported to occur in more than 80 countries, with estimates from the Centers for Disease Control and Prevention suggesting that there are ~476,000 new cases per year in the United States alone (2), where the medical costs associated with the management of Lyme disease and its sequelae approach $1.3 billion annually (3). If detected in the early stage of infection, antibiotics resolve clinical symptoms in most cases. However, the diagnosis of Lyme disease requires serious improvement, especially for the detection of early disease and the discrimination of active versus resolved infections (4). Moreover, despite current full-scale antibiotic therapy, persistent or relapsing symptoms can later develop in a subset of patients, a condition referred to as post-treatment Lyme disease syndrome (5), for which the etiology, pathogenic mechanisms, and treatment remain unknown (6). Despite substantial efforts over several decades to assess protective immunity (7), a human vaccine against Lyme disease is yet unavailable (8); therefore, the identification of novel antigenic targets for the diagnosis and prevention of Lyme disease remains a highly warranted research goal. As whole cell spirochete lysates contain some of the abundant Osps, many of which are irrelevant to mammalian infection (9–11), herein we explored whether their removal would allow the identification of novel yet non-abundant antigens (Fig. 1A), with an ultimate goal of determining whether these non-abundant yet immunogenic antigens could serve as targets for novel diagnostic and preventive strategies for Lyme disease.

### Generation of the *Osp^ABC−^* mutant

To systematically remove OspA, OspB, and OspC from an infectious and clinical isolate of *B. burgdorferi*, we used a previously generated *ospAB* mutant (13) already deficient in OspA and OspB and sought to further delete *ospC* via targeted mutagenesis. The *ospAB* mutant, which is in infectious 297 background (strain AH130) and confers kanamycin and streptomycin resistance (13), was transformed with the suicide vector pCKO for the inactivation of *ospC* (12), conferring resistance to erythromycin. After an overnight recovery, streptomycin and erythromycin were added to the transformation mixture at a final concentration of 50 µg/mL and 50 ng/mL, respectively. The mixtures were then plated into multiple 96-well tissue culture plates at dilutions of 1:1, 1:10, and 1:50. Two weeks after plating, the wells that contained positive cultures were identified by a color change of the medium and verified by dark-field microscopy for the presence of viable spirochetes. The successful inactivation of *ospC* in the *ospAB* mutant was confirmed by gel electrophoresis and real-time quantitative reverse transcription PCR analysis (Fig. 1B) using published procedures (14). Plasmid profiles were performed, and a clone with an identical endogenous plasmid profile with AH130 was then selected for further work, which reflected similar growth kinetics as compared to the parental isolate (Fig. 1C). Although a deficiency of OspA and OspB has been shown to activate the Rrp2-RpoN-RpoS pathway (sigmaN-sigmaS cascade) in *B. burgdorferi* (15), the abrogation of these proteins together with OspC, however, had mixed effects, wherein the expression of some of the tested genes, such as *dbpA*, *dbpB*, *bba07*, and *mlp10*, were differentially expressed in the mutant, while the expression of others, such as *ospF*, *mlp7*, and *mlp8*, remained unaltered (Fig. 1D). These results further underscore the remarkable intricacies of borrelial gene regulation cascades, where the expression of many genes is interlinked, including genes that encode Osps, with possible relevance to spirochete adaptations in the infectious cycle (10, 16, 17).

### Differential detection of proteins in *Osp^ABC−^* mutant

We next used gel electrophoresis and immunoblotting to identify non-abundant cellular and membrane proteins that could be more highly detectable in the *Osp^ABC^*^−^ mutant but otherwise are undetectable or poorly detectable in the wild-type isolate. *B. burgdorferi* cells were grown to an equal cell density and a resolution of 25 µg of total spirochete lysates in a preparatory vertical SDS-PAGE using a longer (16 cm) 5%–20% gradient gel, showing that several protein bands were in higher abundance in the *Osp^ABC^*^−^ mutant (Fig. 1E). Immunoblot analysis for targeted detection of representative sub-surface and surface spirochete proteins, such as FlaB and BmpA (18), showed that both proteins are detectable in the mutant (Fig. 1F). Next, the analysis of Triton-X-114-extractable detergent phase proteins, which mostly represent membrane-associated and hydrophobic proteins (19), displayed their differential detection in the mutant (Fig. 1G). Immunoblot analysis using an antibody generated against *B. burgdorferi* outer membrane vesicle (OMV) (20, 21) showed that OspA, OspB, and OspC were highly detectable in wild-type cells, while neither these Osps nor additional detergent phase proteins were detectable in the mutants (Fig. 1G, rightmost panel), suggesting that borrelial OMV is predominantly composed of these three Osps.

**Fig 2:**
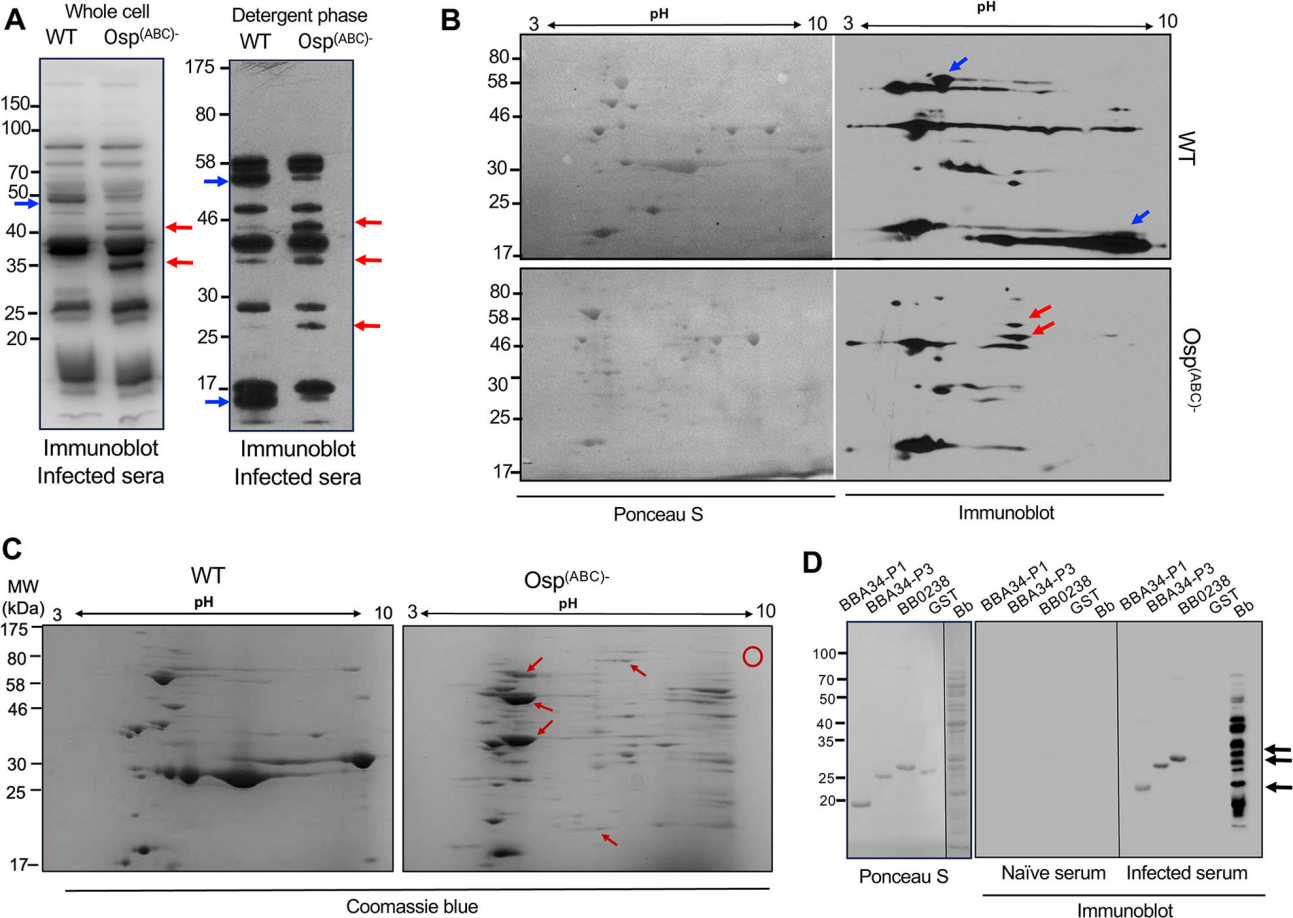
Identification of non-abundant and immunogenic proteins in *Osp^ABC^*^−^ mutant. (**A**) Detection of immunogenic proteins. Whole cell (left image) and Triton-X-114 extractable detergent phase proteins (right image) from the wild type and *Osp^ABC^*^−^ mutants grown at late log phase were immunoblotted with sera collected from mice infected with *B. burgdorferi*. Protein bands that were more detectable in the mutant or wild type cells are indicated by red or blue arrows, respectively. (**B**) Whole cell spirochete lysates, as shown in panel A, were resolved using 2D SDS-PAGE, transferred onto nitrocellulose membrane, stained with Ponceau S (left image), and immunoblotted (right panel) with anti-*B*. *burgdorferi* sera as described in panel A. Protein bands that were more detectable in the mutant or wild type cells are indicated by red or blue arrows, respectively. (**C**) Whole cell spirochete lysates were resolved using 2D SDS-PAGE and stained with Coomassie blue. Protein bands that were more detectable in the mutant cells (representative ones are shown by red arrows) were excised and processed for protein identification using mass spectrometry (MS). A blank area (red circle) was excised and similarly processed for MS analysis as a negative control. (**D**) Identification of BBA34 and BB0238 as immunogenic proteins during *B. burgdorferi* infection in mice. One microgram of the recombinant forms of two truncated BBA34 (P1 and P3), BB0238, and control protein GST (glutathione S-transferase), as well as aliquots of wild-type spirochete lysates (Bb), was separated in 12% gradient SDS-PAGE, transferred onto nitrocellulose membrane, stained with Ponceau S (left image), or immunoblotted with sera from either naïve mice or mice infected with *B. burgdorferi* (middle and right images)*,* as detailed in panel A. The immunoreactivities of BBA34 (P1 and P3) and BB0238 in the infected sera, not in naïve sera, are indicated by arrows.

We next compared the protein profiles between wild type and mutant isolates that are antigenically detectable during *B. burgdorferi* infection of mice. A group of three C3H mice were infected with *Ixodes scapularis*-transmitted *B. burgdorferi*, and serum samples were collected from the animals at 3 weeks after infection using published procedures (14). Immunoblotting of aliquots of whole cell lysates or Triton-X-114-extractable hydrophobic proteins from the wild type and the *Osp^ABC^*^−^ mutant grown at the late log phase (10^8^ cells/mL) revealed that few proteins were differentially detected in the mutant (Fig. 2A). This is consistent with the fact that *B. burgdorferi* downregulates the majority of its immunogenic proteins, such as surface lipoproteins, during late murine infection (9). The separation of whole cell lysates using two-dimensional (2D) gels and subsequent immunoblotting also revealed protein spots that are differentially detectable in the mutant (Fig. 2B). Finally, using a preparatory 2D gel, we excised representative protein spots that were differentially present in the *Osp^ABC^*^−^ mutant (Fig. 2C), which were further processed for protein identification using liquid chromatography-mass spectrometry, as previously described (22). Please note that, while detectable protein spots are grossly similar between 2D gels of *Osp^ABC^*^−^ mutants, there are also differences (Fig. 2B and C), likely due to variations in the precise conditions or growth phases of spirochete cultures, or sample preparations, among other possibilities. Notwithstanding, our analysis identified many spirochete proteins that were consistently and differentially detectable in *Osp^ABC^*^−^ mutants, representing the following functional categories: membrane transporters, proteins of unknown functions, chaperons, and metabolic enzymes (Table S1). Although transcripts for *dbpA*, *dbpB*, *bba07*, and *mlp10* were differentially expressed in the mutant (Fig. 1D), the corresponding proteins were not detected (Table S1), possibly due to disparate technologies that were used to detect mRNA and its translated products in spirochete cells. While a targeted set of mRNAs was measured by PCR, which involved many cycles of nucleic acid amplifications, proteins were detected in a non-targeted fashion by mass spectrometry that lacks any product amplification cycle; in addition, the stability and abundance of a given transcript and its translated product in a cell could also differ, which altogether could contribute to the fact that differentially expressed genes in Fig. 1D are missing in Table S1. Nevertheless, future studies are needed to uncover a more thorough identification of differentially expressed transcripts and proteins at various growth phases of the *Osp^ABC^*^−^ mutant, as compared to the wild type. Notably, some of these proteins, such as BBA34, BB0238, BBB16, BB0328, and BB0383 (Table S1), have been previously suggested to be immunogenic during the natural infection of humans and white-footed mice with *B. burgdorferi* (23). As these proteins also include sub-surface or periplasmic proteins, such as BBA34 (24) and BB0238 (25), we sought to further validate their immunogenicity during spirochete infection in the tick-borne murine model of Lyme borreliosis. Immunoblotting analysis using recombinant forms of truncated BBA34 and BB0238, produced using published procedures (25), and anti-*B*. *burgdorferi* sera collected from infected mice (Fig. 2A) showed that these proteins are immunogenic during experimental murine infection (Fig. 2D).

Collectively, the data presented herein suggest that it is possible to identify non-abundant and apparently sub-surface antigenic markers of *B. burgdorferi* infection by the removal of abundant Osps in spirochetes. A set of differentially expressed transcripts and proteins identified in the Osp-deficient cells allows for future exploration of novel aspects of spirochete gene regulation cascades, which play key roles in pathogen persistence in its enzootic infectious cycle. As some of these proteins are also known to be immunogenic during murine and human infections, their future characterization and use might contribute to a better understanding of *B. burgdorferi* infection biology and the development of diagnostic markers and vaccines against Lyme disease.

## SUPPLEMENTAL MATERIAL

Table S1 msystems.01087-23-s0001.docx Differentially detectable proteins.

ASM does not own the copyrights to Supplemental Material that may be linked to, or accessed through, an article. The authors have granted ASM a non-exclusive, world-wide license to publish the Supplemental Material files. Please contact the corresponding author directly for reuse.
