# Supplementary material for: A systemic approach to identify non-abundant immunogenic proteins in Lyme disease pathogens
Source: mSystems. 2023 Dec 11;9(1):e01087-23. doi: 10.1128/msystems.01087-23 (PMC10805064; doi:10.1128/msystems.01087-23)
Supplement: Table S1 — Differentially detectable proteins. [file msystems.01087-23-s0001.docx]

**Table S1**: **Differentially detectable proteins in Osp^ABC-^-deficient *B. burgdorferi* compared to the wild type isolate**

| **Name/Gene ID*** | **Accession number** | **Description** |
| --- | --- | --- |
| BB_A34/ OppAV | AAC66262.1 | Peptide ABC transporter substrate-binding protein |
| BB_0238 | AAC66635.2 | Conserved hypothetical protein |
| BB_0540 / FusA | AAC66897 | Elongation factor G |
| BB_0536 | AAC66901 | Zinc protease, putative |
| BB_0691 / FusA | AAC67051 | Translation elongation factor G |
| BB_0560 / HtpG | AAC66919 | Chaperone protein HtpG |
| BB_0329 | WP_002656717.1 | Peptide ABC transporter substrate-binding protein |
| BB_B16 / OppAIV | AAC66315 | Oligopeptide ABC transporter OppAIV |
| BB_0328 | AAC66708 | Bacterial extracellular solute-binding protein, family 5 |
| BB_0383 / BmpA | AAC66757 | Basic membrane protein A, immunodominant antigen P39 |
| BB_0502 / RpoA | AAC66840 | DNA-directed RNA polymerase, alpha subunit |
| BB_0158 | AAC66550 | S2 lipoprotein |
| BB_0658 | AAC67007 | Phosphoglycerate mutase family protein, putative |
| BB_0241 / GlpK | AAC66628 | Glycerol kinase |
| BB_0057 / Gap | AAC66450 | Glyceraldehyde-3-phosphate dehydrogenase, type I |
| BB_0055 / TpiA | AAC66452 | Triose-phosphate isomerase |
| BB_0105 / Map | AAC66499 | Methionine aminopeptidase, type I |
| BB_0628 | AAC66985 | Lipoprotein, putative |
| BB_0518 / DnaK | AAC66887 | Chaperone protein DnaK |
| BB_0610 / Tig | AAC66965 | Trigger factor |
| BB_0342 / GatA | AAC66715 | Glutamyl-tRNA(Gln) amidotransferase subunit A (Glu-ADTsubunit A) |
| BB_0264 | AAC66683 | Heat shock protein 70 |
| BB_0059 | AAC66449 | CBS domain pair protein |
| BB_0676 / Gph | AAC67019 | Phosphoglycolate phosphatase |

* Genes listed in the shaded area represent ones that were detectable in at least two independent 2D gels and mass spectrometry analyses; others were inconsistently detected in individual experiments.
